# The research on endothelial function in women and men at risk for cardiovascular disease (REWARD) study: methodology

**DOI:** 10.1186/1471-2261-11-50

**Published:** 2011-08-10

**Authors:** Simon L Bacon, Kim L Lavoie, André Arsenault, Jocelyn Dupuis, Louise Pilote, Catherine Laurin, Jennifer Gordon, Denyse Gautrin, Alain Vadeboncoeur

**Affiliations:** 1 Montreal Behavioural Medicine Centre, Montreal, Canada; 2 Research Centre, Montreal Heart Institute - a University of Montreal affiliated hospital, Montreal, Quebec, Canada; 3 Department of Exercise Science, Concordia University, Montreal, Quebec, Canada; 4 Research Centre, Hôpital du Sacré-Coeur de Montréal - a University of Montreal affiliated hospital, Quebec, Canada; 5 Department of Psychology, University of Quebec at Montreal (UQAM), Montreal, Quebec, Canada; 6 Department of Medicine, Université de Montréal, Montreal, Quebec, Canada; 7 Department of Medicine, McGill University, Montreal, Quebec, Canada; 8 Department of Psychology, McGill University, Montreal, Quebec, Canada

**Keywords:** endothelium, sex, gender, forearm hyperaemic reactivity, cardiovascular disease

## Abstract

**Background:**

Endothelial function has been shown to be a highly sensitive marker for the overall cardiovascular risk of an individual. Furthermore, there is evidence of important sex differences in endothelial function that may underlie the differential presentation of cardiovascular disease (CVD) in women relative to men. As such, measuring endothelial function may have sex-specific prognostic value for the prediction of CVD events, thus improving risk stratification for the overall prediction of CVD in both men and women. The **primary objective **of this study is to assess the clinical utility of the forearm hyperaemic reactivity (FHR) test (a proxy measure of endothelial function) for the prediction of CVD events in men vs. women using a novel, noninvasive nuclear medicine -based approach. It is hypothesised that: 1) endothelial dysfunction will be a significant predictor of 5-year CVD events independent of baseline stress test results, clinical, demographic, and psychological variables in both men and women; and 2) endothelial dysfunction will be a better predictor of 5-year CVD events in women compared to men.

**Methods/Design:**

A total of 1972 patients (812 men and 1160 women) undergoing a dipyridamole stress testing were recruited. Medical history, CVD risk factors, health behaviours, psychological status, and gender identity were assessed via structured interview or self-report questionnaires at baseline. In addition, FHR was assessed, as well as levels of sex hormones via blood draw. Patients will be followed for 5 years to assess major CVD events (cardiac mortality, non-fatal MI, revascularization procedures, and cerebrovascular events).

**Discussion:**

This is the first study to determine the extent and nature of any sex differences in the ability of endothelial function to predict CVD events. We believe the results of this study will provide data that will better inform the choice of diagnostic tests in men and women and bring the quality of risk stratification in women on par with that of men.

## Background

### Endothelial function: a potentially novel predictor of cardiovascular disease (CVD) risk in men and women

The endothelium is the largest endocrine organ in the human body and is involved in the control of vascular tone, platelet reactivity, coagulation, and permeability [1]. As such, a healthy endothelium protects against excessive/abnormal inflammation and coagulation [2], which are key processes in CVD development and progression. The transition from a normal to a dysfunctional endothelium is associated with abnormal vasomotor activity, the development of a pro-coagulant surface, and an acceleration of the inflammation process [1]. As such, loss of normal endothelial function is a key event in the initiation and progression of atherosclerosis [3].

It has been suggested that the association between endothelial dysfunction and classical risk factors for atherosclerosis (e.g., age, diabetes, hypertension, smoking, dyslipidemia) [4] supports the concept that endothelial *dysfunction *may be regarded as "an integrated risk of risk factors", indicating that it could serve as a highly sensitive marker for the overall cardiac risk of an individual [5]. In addition, a recent review found that patients with endothelial dysfunction had a 3.5 (range 2.5 - 5.0) greater chance of having a cardiac event compared to those with normal endothelial function [4]. Taken together, these findings suggest that measuring endothelial function has good prognostic value for the prediction of CVD events, and may be superior to more traditional methods of risk stratification (e.g., Framingham Risk Score). This potential utility is underscored by the fact that in many cases, traditional methods for stratifying cardiac risk in individuals are poor. For example, one study including over 12,000 men and women from Scotland reported that the Framingham Risk Score significantly underestimated cardiovascular mortality [6]. In addition, both the Framingham Risk Score and the National Cholesterol Education Program (NCEP) model have been shown to generally *underestimate *CVD risk in women [7-10]. Moreover, the Framingham Risk Score has been shown to *overestimate *CVD risk in men, particularly among non-American cohorts [11,12]. Similar limitations have been found with other risk models (e.g., the Reynolds Risk Score) [13,14]. As such, there is clearly a need for better prediction tools that provide more reliable estimates in men and women.

### Sex differences in endothelial function

There is evidence of important sex differences in endothelial function, many of which may underlie the differential presentation of CVD in women relative to men [15-17]. Women have greater microvascular dysfunction relative to men [18], which seems to be important in the aetiology of endothelial dysfunction. Moreover, both chest discomfort and chest pain in the absence of overt coronary artery disease have been linked to greater endothelial dysfunction in women [19]. There is also evidence of direct sex hormone effects on endothelial function. For example, the menstrual cycle has been shown to modulate endothelial function in healthy women. Specifically, studies have indicated that there is an increase in endothelium-dependent vasodilation as women pass from the follicular phase to the luteal phase [20,21], a period associated with an estrogen surge [21]. In comparison to healthy, age-matched men, healthy women tend to have better endothelial function, which may be due to improvements seen in both the follicular and luteal phases (compared to menses) [22]. However, it should be noted that another study found that brachial artery endothelium-dependent vasodilation (as measured by ultrasound) was better in women compared to men, but this was largely dependant on basal brachial artery diameter rather than hormonal effects [23].

Further evidence of a link between sex, endothelial function, and CVD is highlighted by the fact that the earlier onset of CVD in men relative to women mirrors a similar pattern for endothelial function, with age-related endothelial dysfunction also occurring earlier in men than women [24,25]. However, around the time of menopause, women have a steep decline in endothelial function such that their endothelial deterioration rapidly catches up with that of men [26,27]. Finally, estrogen, progesterone, and androgen receptors have all been found in abundance in human vascular endothelium, with specific sex differences in the expression of hormone receptors [28]. Not only does hormone replacement therapy directly stimulate endothelial receptors, but also indirectly modifies molecules known to affect the endothelium, e.g., lipoproteins, homocysteine [29].

Though it has been suggested that endothelial function has good prognostic value for the prediction of a broad range of important CVD events [4], to our knowledge, no studies to date have specifically assessed sex differences in the prognostic value of endothelial function testing. If there are sex-specific differences in the processes underlying risk factor injury and atherosclerotic responses that are responsible for the unique presentation of CVD in women, measuring endothelial function may help elucidate these differences. It may also help improve risk stratification and the overall prediction of CVD in both men and women.

### Current measures of endothelial function

There are currently no direct measures of endothelial function. However, there are several indirect ways to estimate endothelial function, most notably using invasive coronary artery testing, the examination of blood markers, and via non-invasive peripheral artery assessments [30]. Due to their relative ease of administration, i.e., use of non-invasive procedures, non-invasive arterial methods are the most frequently used and include plethysmography, ultrasound imaging, near infrared spectrometry, and transcutaneous oximetry. Although the optimal method of assessing endothelial function is still under debate [31], ultrasound imaging of the brachial artery during reactive hyperaemia (i.e., increased blood flow) [32] is the most popular techniques. This method, called flow-mediated dilatation, measures vascular reactivity in response to hyperaemic challenge as its proxy measure of endothelial function. Although it is considered the current 'gold standard,' assessment of flow mediated dilatation poses several important challenges. In order to have adequate within laboratory reproducibility, administering this test requires very specialised training for the ultrasound technician, and even with extensive training, there is generally poor between laboratory reliability [33]. These limitations have meant that flow mediated dilatation has had limited clinical utility [34]. The current study addresses these limitations by employing a novel method of assessing brachial artery reactivity (as a proxy measure of endothelial function) which has high reproducibility and reliability (see below for details) [35].

#### Study Objectives, Aims and hypotheses

The **primary objective **of this study is to assess the clinical utility of brachial artery reactivity (a proxy measure of endothelial function) for the prediction of CVD events in men vs. women using a novel, noninvasive, nuclear medicine-based approach based on the reactive hyperemic response.

This study has the following specific aims:

##### Primary aim

The primary aim is to prospectively evaluate sex differences in the extent to which endothelial function at baseline predicts risk for CVD events (including cardiac mortality, non-fatal myocardial infarction (MI), percutaneous coronary intervention's (PCI's), coronary artery bypass graft surgery (CABG), and cerebrovascular events) 5 years after a dipyridamole myocardial perfusion imaging (MPI) test.

##### Hypotheses

1) Endothelial dysfunction (as measured by brachial artery reactivity) will be a significant predictor of CVD events independent of baseline stress test results, clinical, demographic, and psychological variables in both men and women; and 2) endothelial dysfunction will be a better predictor of events at 5 years in women compared to men.

##### Secondary aim

To evaluate the prognostic value of endothelial function testing compared to existing risk measures (i.e., pharmacological (dipyridamole) testing and the Framingham Risk Score) in men vs. women for the prediction of CVD events.

##### Hypotheses

1) Endothelial function testing will demonstrate greater sensitivity and specificity for the prediction of CVD events at 5 years than the other risk measures; 2) Endothelial function testing will show greater sensitivity and specificity for the prediction of CVD events at 5 years in women compared to men; and 3) Endothelial function testing will add significant incremental prognostic value to the other risk measures for the prediction of CVD events.

Additional exploratory analyses will be conducted to assess associations between various measures of psychological stress (e.g., anxiety, depression) and health behaviours (e.g., smoking, obesity, and physical activity) and endothelial function, and interactions between these variables with sex/gender. This is based on evidence suggesting that endothelial function may be impacted by both psychological and behavioural factors, including depression, smoking, and obesity, and that there may be a sex-specific nature to these relationships [36-39].

## Methods/Design

### Patient Selection

**Figure 1:**
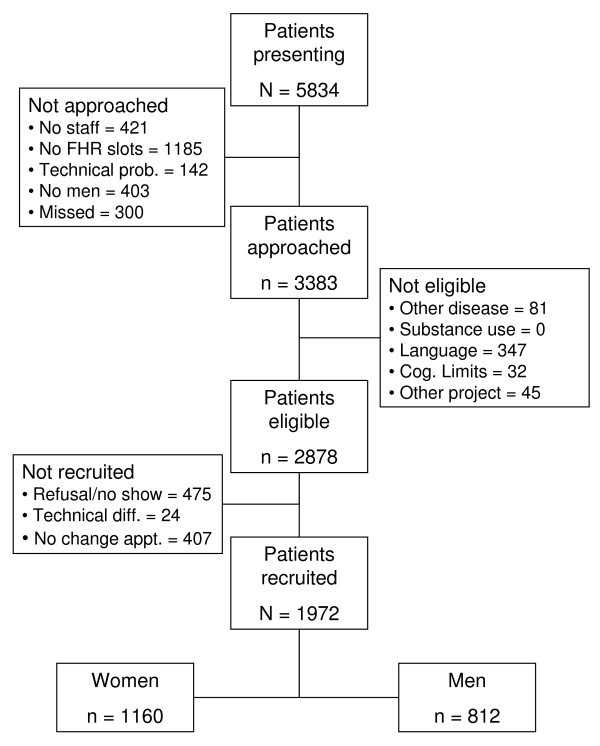
**Baseline Study Recruitment**. (TO APPEAR AT THE FOOT OF THE TABLE) For those not approached, No staff = there were no staff available for those patients; No FHR slots = all the slots were taken for the days testing; Technical prob. = there were technical problems with the SPECT camera's and MPI testing was cancelled for that time; No men = we were not recruiting men at certain points to ensure an over sampling of women; Missed = the research assistant was too busy with other participants to check these dossiers.

A total of 1972 patients (812 men and 1160 women) undergoing a dipyridamole stress single photon emission computer tomography (SPECT) study in the Nuclear Medicine Department of the Montreal Heart Institute were recruited to undergo the baseline assessment. Recruitment was carried out between January 2007 and December 2010. As detailed in Figure 1, a total of 5822 patients presented to the department for dipyridamole stress testing, of which 3383 (58%) were screened for inclusion in the study. A total of 505 patients were excluded. Of the remaining 2878 eligible patients, 475 (17%) refused to participate or did not show-up for their appointment, 407 (14%) could not change their appointment to accommodate the testing schedule, and 24 (1%) were not tested due to SPECT equipment failure on the day of testing. This left a final participating sample of 1972 patients, which was predominantly white (95%) and for which the average (SD) age was 67 (10) years for the men and 67 (10) years for the women. Based on previous data collected by the investigators and use of similar follow-up techniques [40], an approximate 15% attrition rate is expected, which would result in a total follow-up sample of around 1700 individuals.

### Baseline Inclusion/Exclusion Criteria

To be included in the study, patients must have undergone an independent, medically-indicated (prescribed) dipyridamole stress SPECT study, and be 18 years of age or older. Patients were excluded if they (1) had a serious co-morbid medical condition for which the patient was not expected to survive the next 12 months (e.g., chronic obstructive pulmonary disease, cancer, other autoimmune disease); (2) smoked or consumed caffeine or xanthine within 6 hours of the endothelial function test; (3) were unable to speak English or French; (4) had severe psychopathology (e.g., schizophrenia), current substance abuse, or apparent cognitive deficits (e.g., dementia) that would render the patient unable to provide informed consent; (5) were currently pregnant; or (6) they were participating in another study which prohibited their involvement in the current study.

### Study Design

#### Baseline

Patients were pre-screened to verify eligibility by a trained clinical research assistant on the first day of a 2-day SPECT study protocol when they presented to the nuclear medicine department. On the day of their rest perfusion image and following the injection of the Tetrofosmin, eligible and consenting patients underwent the forearm hyperemic reactivity (FHR) test (a nuclear medicine based proxy measure of endothelial function, see below) [35]. Sociodemographic, medical, clinical (including menopause status and menstrual cycle phase in women), and psychological data were collected across the two days of the stress test protocol via structured interview and self-report questionnaires. In addition, the prevalence of depressive and anxiety disorders was assessed using a brief structured psychiatric interview - the Primary Care Evaluation of Mental Disorders (PRIME-MD).

#### Follow-up

**Figure 2:**
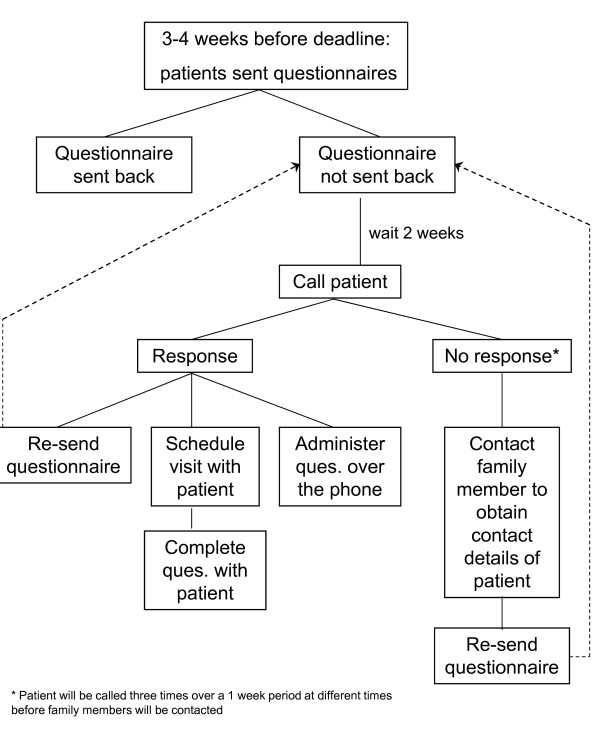
**Details of follow-up procedure for years 1 and 3**.

**Figure 3:**
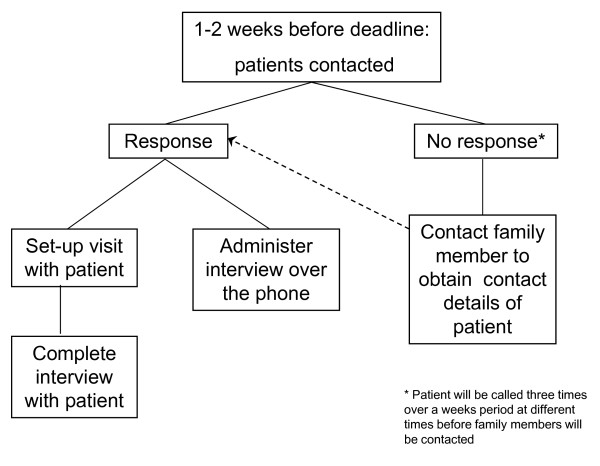
**Details of the follow-up procedure for year 5**.

All patients will be re-contacted one, three, and five years after enrolment to ascertain self-reported CVD events and to determine changes in medical, behavioural, and psychological status. These data will be collected via questionnaire that will be mailed to participants with a pre-addressed stamped envelope for returning the information (see Figures 2 and 3 for the follow-up algorithms). In addition, at the 5-year follow-up assessment, patients will have the PRIME-MD re-administered in person or by phone. All events occurring over the 5-year follow-up will be confirmed (including death) by reviewing hospital medical and provincial electronic database (i.e., Régie de l'assurance maladie du Québec [RAMQ], MedEcho, and the Institut de la statistique du Québec [ISQ]) records. This study was approved by the Research Ethics Board of the Montreal Heart Institute and all patients provided written, informed consent.

### Baseline Measures

#### Dipyridamole stress test study

The dipyridamole test was carried out according to standard protocol [41]. The rest image was collected on day 1 and the dipyridamole stress image on day 2. For the dipyridamole study, a total dose of .568 mg/kg of dipyridamole diluted in a 20 ml saline was infused at a regular rate over a period of 4 min. A maximum dose of 50 mg was used. A dose of 15 to 22 mCi of the radioactive tracer (Technetium Tc-99 m Tetrofosmin) was injected 6 min after the administration of dipyridamole. Heart rate was recorded by electrocardiogram (ECG: and electronically stored) at baseline, during every minute of the test, and during recovery. Blood pressure was recorded every 1-3 min from baseline through to the end of recovery. During recovery, aminophylline and/or nitroglycerine was administered at the discretion of the attending physician. Approximately 45 min after the end of the test, the patient had their SPECT image recorded. On the rest day, the patient was injected with Technetium Tc-99 m Tetrofosmin during the endothelial function test, and had their SPECT image recorded approximately 45 min later.

SPECT images were obtained using a triple-head, large field of view camera equipped with low-energy, high-resolution, parallel-hole collimators. Sixty-four projections were acquired over a 360^o ^variable elliptic orbit on a 64 × 64 × 16-byte matrix with a zoom of 1.44. A 20% symmetric energy window centered on the 140 keV peak was used. Processing will be performed using filtered back-projection with a Butterworth filter (cutoff 0.7, order 8) without attenuation correction. Short-axis slides, each one pixel thick (5.3 mm), will be reconstructed and displayed as a two-dimensional polar map. Standard software (Autoquant) estimates of stress, rest, and stress-rest differential scores will be used to define reversible stress-induced abnormal images (i.e., ischemia).

#### Assessment of endothelial function using the Forearm Hypereamic Reactivity (FHR) test

Our technique for assessing endothelial function is a nuclear medicine variation of the well-established flow-mediated dilation protocol [35,42-44]. The approach is based on the intravenous injection of a tracer and the simultaneous non-invasive external detection of the tracer ingress and transit into a forearm submitted to reactive hyperaemia and the control contralateral forearm. Approximately 1 hr before the rest SPECT image, a hyperemic challenge was administered by the nuclear technician by inflating a blood pressure cuff placed on the right arm 50 mm Hg above systolic blood pressure for 5 minutes. The patient was seated with both arms extended over the top of large field of view gamma-camera. Thirty seconds after sudden cuff release, the Technetium Tc-99 m Tetrofosmin was injected as a bolus. A dynamic (1 frame/second) image of the forearms was recorded for 5 minutes. The standard SPECT rest imaging was carried out approximately 30-45 minutes after the completion of testing. The specific measure of the FHR test which is used as a proxy of endothelial function, the Rate of Uptake Ratio (RUR), is derived by comparing the first-pass activity-time curves between the hyperaemic and non-hyperaemic arms. In our previous research, we found that an RUR of 3.55 discriminates between patients with and without coronary artery disease (CAD), i.e., patients with a RUR ≤ 3.55 were more likely to have CAD [35]. It is believed that a higher RUR may be indicative of better endothelial function (i.e., greater uptake in the hyperaemic arm compared to the control arm). However, it should be noted that whilst RUR is partly dependent on endothelial function (as per flow-mediated dilatation), it is also probable that there are other more global vascular factors which influence this too.

The FHR technique offers a number of advantages over existing tests. It is a non-invasive technique that allows for repeated measurements with an internal control (non-hyperaemic arm) and discriminates between CAD and non-CAD patients with a high degree of sensitivity (95%) and specificity (90%) [42]. It is easy to administer and minimizes human error in administration of the test, is highly reproducible (intra-class r = .89) [43], and shows high (r = .98) inter-rater reliability [45]. In addition, it has the potential to be easily administered in any centre with nuclear medicine facilities. In contrast, the need for such facilities also limits its wider usage in research and general medical settings (e.g., primary care).

#### Demographic, Medical, and Health Behaviour Assessment

The following information was collected via structured interview from each patient at baseline: age, ethnicity, socioeconomic status (years of education, income, occupation [46], and residential deprivation [47,48]), marital status, current and lifetime history of CVD, CVD risk factors [e.g., hypertension, hyperlipidemia], co-morbid conditions, medications (including hormone replacement therapy in women), details of immediate and extended family history of CVD, levels of physical activity [49,50], and current and lifetime history of smoking and alcohol use/abuse [51]. In addition, patients' height, weight, and waist circumference (WC) were measured on the day of the stress test to calculate body mass index (BMI) and central adiposity.

#### Psychiatric Disorders, psychological status, quality of life, and gender identity

To assess depressive and anxiety disorders (which are the most common psychiatric disorders seen in cardiac populations) [52,53], patients underwent the mood and anxiety disorders modules of the Primary Care Evaluation of Mental Disorders (PRIME-MD [54]). The PRIME-MD is a brief, psychiatric interview that generates psychiatric diagnoses based on Diagnostic and Statistical Manual of Mental Disorders criteria. It takes 5-15 minutes to administer and score, and yields diagnoses of comparable sensitivity and specificity for individual disorders (e.g., major depression: sensitivity (83%) and specificity (88%) as longer structured interviews [54]. Participants also completed a battery of self-report psychological questionnaires including the Anxiety Sensitivity Index [55], the Beck Anxiety Inventory [56], the Beck Depression Inventory-II [57], the Whiteley Hypochondriasis Scale [58], the Multidimensional Health Locus of Control Scale [59], the Cook-Medley Hostility Inventory [60], and the Short Form 36 quality of life scale [61]. Patients also completed the Bem Sex Role Inventory [62] which measures gender identity (masculinity and femininity).

#### Blood-Based Measures

On the day of the rest perfusion study and once the catheter had been sited for the tracer injection, a blood sample was drawn by the nuclear technician to obtain biochemical measures. Fasting measures of C-reactive protein, creatinine, uric acid, sedimentation, Haemoglobin A1C, glucose, triglycerides, insulin, a lipid panel (total cholesterol, high density lipoproteins, and low density lipoproteins), and a full blood count were obtained. In addition, we obtained measures of lutenizing hormone (LH), follicular stimulating hormone (FSH), estrogen, and testosterone in both men and women.

#### Framingham Risk Score Calculation

The Framingham risk score will be determined for each patient using medical and clinical data collected during the baseline assessment. Separate risk scores for each model will be calculated for men and women using the algorithms published by Wilson et al. [63]http://www.framinghamheartstudy.org/risk/coronary.html.

### Follow-up Measures

#### Events Assessment

At the end of the study (an average of 5 years post recruitment of the baseline sample), all billable events and diagnostic procedures occurring in the province of Quebec will be obtained from the Commission de l'accés à l'information du Québec (the Quebec access to information commission). Patients and events will be linked using a unique identifier (the numéro d'assurance maladie: medical insurance number). Using the same identifier, mortality data (including date and cause of death) will be collected from the ISQ (Quebec's statistic institute). Using a standard protocol for the definition of CVD events [64], the primary study endpoint will be defined as a combination of cardiac mortality, non-fatal MI, revascularization procedures, and cerebrovascular events (i.e., stroke). In addition, **s**elf-reported information regarding medical status and medications, as well as all procedures and events undertaken since the last follow-up contact will be collected.

#### Health behaviours

Information regarding changes in all health behaviours assessed at baseline (e.g., tobacco and alcohol consumption, physical activity level) will be collected. Self-reported weight and WC will also be obtained to assess changes in BMI and WC.

#### Psychological variables, quality of life, and gender role

To assess changes in psychological variables, patients will complete the same self-report questionnaires as at baseline. Also, at the final (5-year) assessment, patients will undergo the PRIME-MD in person or by phone to verify changes in psychiatric status.

### Statistical Analyses

#### Primary aim

To test the main hypothesis, Cox proportional hazards models will be used to evaluate the effect of sex and endothelial function (independent binary variable, defined using our previously published cut-point of 3.55 [35]) on the time of occurrence of composite CVD events. Fixed covariates will be derived from baseline stress test, clinical, demographic, and psychological characteristics using Harrell's guidelines [65]. The models will be validated for calibration and discrimination using standard bootstrapping techniques [65]. In addition, we will estimate the concordance statistic (c-statistic), which represents the probability that for a randomly selected pair of individuals, one diseased and one non-diseased, the diseased individual has the higher estimated disease probability [66].

#### Secondary aims

To test the hypothesis that endothelial function will have higher specificity and sensitivity than other risk stratifiers (dipyridamole test and Framingham Risk Score) in the prediction of CVD events, a series of receiver operating characteristic (ROC) curve analyses will be used, adjusting for covariates. Initially, ROC curve analyses will be conducted individually for endothelial function and the dipyridamole test or the Framingham Risk Score, and the sensitivities and specificities will be compared. Following this initial step, a final ROC curve will be run where endothelial function and SPECT or the Framingham Risk Score will be entered simultaneously, which will allow for the determination of the additive value of endothelial function. This data will allow us to estimate both the positive and negative predictive value of endothelial function. Three separate series of ROC curve analyses will be used, one for men, one for women, and one where both sexes are included. Due to the extensive and varied number of tests, multiple tests will be corrected with Benjamini and Hochberg's False Discovery Rate correction procedure [67-69]. All analyses will be conducted using SAS (SAS Inc, NC).

### Power calculations

Power calculations were performed to detect significant differences in the rate of CVD events identified in the primary aim between women and men and between those with normal RUR scores and those with reduced RUR scores (i.e., endothelial dysfunction). According to published data [70-72], we estimated an overall rate of CVD events for women of 22% during the total follow-up period. The equivalent estimate for men was 42% [71,72]. Due to a dearth of data in women and a desire to develop a solid database for future exploratory analyses, we decided to over sample women in the current study, such that we would have 1200 (60%) women and 800 (40%) men. Given this sample size, combined event rates over the course of the follow-up are expected to be 30%. Adjustment of the sample size for an anticipated loss to follow-up of 15% would leave a final sample size of 1700 (1020 women and 680 men). In a multivariate Cox regression analysis of the hazard ratio(log) of CVD mortality on the predictor variable 'endothelial dysfunction' with an estimated SD of 0.48, a 0.05 significance level (two-sided), a variance inflation factor of 0.2 for the sex interaction [73], and an R^2 ^adjustment of 0.5 for covariates, setting power at 0.9 would mean that a hazard ratio of 1.53 would be detectable. It should be noted that these estimates are conservative and that a recent review of the effects of endothelial function on CVD outcomes found that those patients with endothelial dysfunction had about a 3.5 (range 2.5 - 5.0) greater likelihood of having a hard CVD event compared to patients without [4]. It also should be noted that as no previous study has assessed the interaction of sex and endothelial function, this more conservative estimate is an appropriate first strategy. All sample size calculations were conducted using the PASS system of NCSS [74].

## Discussion

### Limitations

Though the study has the potential to answer a number of important clinical questions, there are several limitations that should be noted. First, the selection of participants was not random and participants were predominantly white. As such, the results may not generalise to all populations. Second, the recruitment of patients undergoing dipyridamole stress tests implies that this study population is a high risk one relative to those undergoing standard exercise stress tests. As such, the risk scores generated from the study may not be used to generate risk estimates for individuals at low to moderate risk of CVD. Third, the FHR test was conducted using the resting SPECT isotope injection. Whilst this has the benefit of reducing unnecessary exposure to additional doses of radiation and lowers cost, it does not allow us to test the independent (from SPECT) application of the FHR test in a clinical setting. It is anticipated that if the REWARD study provides positive (prognostic) results, that future studies will assess the cost-effectiveness of a stand-alone FHR test in a clinical setting, noting the potential for greater radiation exposure and potential patient safety concerns.

### Originality, innovativeness, and importance

This is the first study to determine the extent and nature of any sex/gender differences in the ability of a novel, nuclear-medicine based measure of endothelial function to predict CVD events that holds great clinical potential for use in cardiac risk stratification of men and women. This is also the largest study to date to directly compare the predictive ability of endothelial function relative to existing risk models (e.g., dipyridamole test and the Framingham Risk Score). We believe the results of this study will provide data that will better inform the choice of diagnostic tests in men and women and bring the quality of risk stratification in women on par with that of men.

## List of Abbreviations

BMI: Body mass index; CABG: Coronary Artery Bypass Graft Surgery; CAD: Coronary Artery Disease; CVD: Cardiovascular disease; FHR: Forearm Hypereamic Reactivity; ISQ: Institut de la statistique du Québec; MI: Myocardial Infarction; MPI: Myocardial Perfuction Imaging; NCEP: National Cholesterol Education Program; PCI: Percutaneous Coronary Intervention; PRIME-MD: Primary Care Evaluation of Mental Disorders; RAMQ: Régie de l'assurance maladie du Québec; ROC: receiver operating characteristic; RUR: Rate of Uptake Ratio; SPECT: Single photon emission computer tomography; WC: Waist circumference.

## Competing interests

André Arsenault owns the patent rights for the FHR procedure (US 64,449,945 B1) and also owns 100% of SyGeSa Ltd who owns the rights for the proprietary software used in the calculation of RUR.

The other authors declare that they have no competing interests.

## Authors' contributions

SLB and KLL conceived of the study, obtained funding for the study, participated in its design and coordination, and drafted the manuscript. AA carried out all FHR assessments and participated in the design of the study. JD provided guidance on the outcomes assessments and participated in the design of the study. LP participated in the design of the study, especially around issues of gender and sex. CL coordinated the data collection and provided input on the psychological measures included. JG participated in data collection and provided input on the psychological measures included. DG provided guidance on the statistical analyses and participated in the design of the study. AV provided medical coverage for all baseline assessments. All authors read and approved the final manuscript.

## Pre-publication history

The pre-publication history for this paper can be accessed here:

http://www.biomedcentral.com/1471-2261/11/50/prepub
